# Lenalidomide Maintenance and Measurable Residual Disease in a Real-World Multiple Myeloma Transplanted Population Receiving Different Treatment Strategies Guided by Access to Novel Drugs in Brazil

**DOI:** 10.3390/cancers15051605

**Published:** 2023-03-04

**Authors:** Anna Beatriz dos Santos Salgado, Roberto Jose Pessoa Magalhães, Robéria M. Pontes, Eduarda da Silva Barbosa, Juan Flores-Montero, Luzalba Sanoja-Flores, Marcelo Gerardin Poirot Land, Glicinia Pimenta, Hélio dos Santos Dutra, Elaine S. Costa, Alberto Orfao, Angelo Maiolino

**Affiliations:** 1 Internal Medicine Postgraduate Program, Faculty of Medicine, Department of Internal Medicine, University Hospital Clementino Fraga Filho, Federal University of Rio de Janeiro (UFRJ), Rio de Janeiro 21044-020, Brazil; 2 University Hospital Clementino Fraga Filho, Federal University of Rio de Janeiro (UFRJ), Rio de Janeiro 21941-617, Brazil; 3 Translational Research Laboratory, Children’s Hospital of Brasilia Jose Alencar, Brasilia 70684-831, Brazil; 4 Fundação Bio-Rio, Federal University of Rio de Janeiro, UFRJ, Rio de Janeiro 21941-599, Brazil; 5 Cytometry Service, Institute of Paediatrics and Puericultura Martagão Gesteira (IPPMG), Faculty of Medicine, Federal University of Rio de Janeiro UFRJ, Rio de Janeiro 21941-912, Brazil; 6 Translational and Clinical Cancer Research Program, Centro de Investigación del Cáncer (CIC-IBMCC-CSIC/USAL), Department of Medicine, Universidad de Salamanca, 37007 Salamanca, Spain; 7 Centro Investigación Biomédicaen Red en Cáncer (CIBERONIC code CB//0040) of Instituto de Salud Carlos III Ministry of Science and Innovation, 28029 Madrid, Spain; 8 Institute of Biomedicine of Seville, Department of Hematology of the University Hospital Virgen del Rocío, University of Seville, 41013 Seville, Spain; 9 Centro de Investigación Biomédica en Red de Cáncer (CIBERONC) Number: CB16/12/00480, Instituto Carlos III, 28029 Madrid, Spain; 10 Institute of Biomedical Science, Federal University of Rio de Janeiro (ICB/CCS/UFRJ), Rio de Janeiro 21941-590, Brazil; 11 Instituto Americas de Ensino e Pesquisa, Rio de Janeiro 22775-001, Brazil

**Keywords:** multiple myeloma, measurable residual disease, lenalidomide, drug access, autologous transplant, maintenance, real-world study

## Abstract

**Simple Summary:**

Lenalidomide maintenance (M-Len) after autologous stem cell transplantation (ASCT) improved survival outcomes in multiple myeloma (MM). The present work found that M-Len and measurable residual disease detected by next-generation flow cytometry (NGF) were independent prognostic factors that could be used to discriminate patients at an earlier risk of relapse in a real-world study from Brazil.

**Abstract:**

Despite recent advances in multiple myeloma (MM), the incorporation of novel agents and measurable residual disease (MRD) monitoring in low-income countries remains a challenge. Although lenalidomide maintenance (M-Len) after autologous stem cell transplantation (ASCT) has been associated with improved outcomes and MRD has refined the prognosis of complete response (CR) cases, until now, there have been no data on the benefits of these approaches in Latin America. Here, we evaluate the benefits of M-Len and MRD using next-generation flow cytometry (NGF-MRD) at Day + 100 post-ASCT (*n* = 53). After ASCT, responses were evaluated based on the International Myeloma Working Group criteria and NGF-MRD. MRD was positive in 60% of patients with a median progression-free survival (PFS) of 31 months vs. not reached (NR) for MRD-negative cases (*p* = 0.05). The patients who received M-Len continuously had a significantly better PFS and overall survival (OS) than those without M-Len (median PFS: NR vs. 29 months, *p* = 0.007), with progression in 11% vs. 54% of cases after a median follow-up of 34 months, respectively. In a multivariate analysis, MRD status and M-Len therapy emerged as independent predictors of PFS (median PFS of M-Len/MRD^−^ vs. no M-Len/MRD^+^ of NR vs. 35 months, respectively; *p* = 0.01). In summary, M-Len was associated with improved survival outcomes in our real-world MM cohort in Brazil, with MRD emerging as a useful reproducible tool to identify patients at an earlier risk of relapse. The inequity in drug access remains a hurdle in countries with financial constraints, with a negative impact on MM survival.

## 1. Introduction

Recent advances in the treatment of multiple myeloma (MM) based on the combination of new drugs and autologous stem cell transplantation (ASCT) have led to improved response rates and survival outcomes [1]. For instance, bortezomib associated with lenalidomide (Len) and steroids for induction therapy, followed by continuous Len maintenance (M-Len), is currently recommended as a standard of care in MM [2]. In different studies, this strategy achieved higher rates of a very good partial response (VGPR)/complete response (CR) associated with a lower percentage of measurable residual disease (MRD)-positive (MRD^+^) cases, and it achieved higher survival rates, with an acceptable toxicity profile [3,4,5].

Although the achievement of CR has traditionally been pursued as the first goal of MM treatment, it has been repeatedly demonstrated that it is a suboptimal surrogate marker of patient progression-free survival (PFS) and overall survival (OS). Thus, CR is associated with heterogeneous outcomes, hiding a large proportion of patients that will not achieve long-term disease control and that will relapse shortly after therapy [6]. In this regard, highly sensitive MRD monitoring has become critical to improving the assessment of the response to therapy in MM, particularly among patients that reach CR or VGPR [7,8]. Indeed, a large number of studies based on different techniques and distinct sensitivity thresholds, including two meta-analyses, have shown that MRD is among the most powerful independent predictors of survival in MM [9,10], with the persistence of residual clonal plasma cells (cPCs) being consistently associated with an inferior PFS [11,12]. In 2016, the International Myeloma Working Group (IMWG) established new response criteria for MM based on the bone marrow (BM) MRD status, evaluated by using eight-color next-generation flow cytometry (NGF) or next-generation sequencing (NGS) reference techniques capable of achieving a sensitivity of <10^−5^ [13].

Due to the economic constraints in Brazil, as well as in other Latin American countries (LATAMC), access to new drugs and all standard routine MM diagnostic and follow-up examinations, including serum electrophoresis, immunofixation, free light-chain determinations and NGF or NGS MRD measurements, is still lacking and/or restricted to reference centers [14]. In turn, the co-existence of dual (i.e., public and private) healthcare systems supported locally by different health insurances leads to the use of unique combinations of first-line therapeutic regimens and laboratory diagnostic and monitoring assays in MM, depending on the specific healthcare system that the patient has access to. As an example of such treatment scenarios in Brazil, the majority of patients eligible for MM transplant in public institutions have access to induction regimens with cyclophosphamide/thalidomide/dexamethasone (CTD), whereas in private centers, bortezomib/cyclophosphamide/dexamethasone (VCD) are preferentially used [14,15,16]. Until recently, most patients received thalidomide or no maintenance after ASCT; however, since Len approval, this drug has become available to patients enrolled in the private (but not the public) healthcare system in Brazil.

Despite all the above, at present, there are no data concerning the potential benefit of introducing M-Len into our current practice or its impact in real-world patients with MM, except for the survival benefits already demonstrated in the pivotal randomized clinical trials used for the approval of this drug [17]. In addition, so far, no data from LATAMC have been reported in which NGF-MRD techniques have been used in addition to conventional response criteria in order to compare local treatments administered in different healthcare conditions/systems within the same country.

In this study, we investigate the impact of continuous M-Len therapy after ASCT and MRD monitoring by carrying out NGF-MRD at Day + 100 after ASCT and identifying subgroups of patients with distinct outcomes among a series of 53 real-world patients with MM treated outside clinical trials in Brazil.

## 2. Material and Methods

Patients and samples: Peripheral blood (PB), BM and 24 h urine samples were collected one hundred days after ASCT (Day + 100) from 53 patients with MM (26 males and 27 females, with a median age of 58 years, ranging from 40 to 70 years), diagnosed according to the IMWG criteria [18] (Table 1). The patients treated in the Brazilian public healthcare system received an MM-oriented treatment fully funded by the government [15], which consisted of six cycles of induction therapy—cyclophosphamide, 300 mg/m^2^; dexamethasone, 40 mg (Day 1, Day 8, Day 15, Day 22); and thalidomide, 100 mg/day (CTD)—followed by ASCT and post-transplant consolidation with two additional cycles of CTD and maintenance with thalidomide (100 mg/day for 10 months), except for patients who suffered from neuropathy. The patients enrolled in the private healthcare system were supported by health insurance companies, with most having access to newly approved therapies and exams, generally restricted to individuals above poverty levels or higher-income employees [15]. This latter group received 4 cycles of bortezomib (1.3 mg/m^2^ SC), cyclophosphamide (300 mg/m^2^) and dexamethasone (40 mg) on Day 1, Day 8, Day 15 and Day 22 (VCD), followed by ASCT and 2 additional consolidation cycles of VCD, followed by M-Len until progression. In both groups, ASCT was performed with PB hematopoietic stem cells mobilized with a granulocyte-colony-stimulating factor (G-CSF). The conditioning regimen consisted of melphalan 200 mg/m^2^ (or 140 mg/m^2^ in patients with renal insufficiency). None of the patients received bortezomib maintenance.

Response assessment: To assess the conventional response to therapy vs. disease progression, all patients from both treatment groups were uniformly evaluated using the IMWG response criteria, based on electrophoresis, immunofixation (IF) in serum and urine and serum free light-chain (sFLC) measurements [13]. CR was defined as the absence of an M-component isotype using IF and <5% PC in BM, and stringent CR (sCR) was defined as the case in which the sFLC ratio values were within the normal range (0.26 to 1.65 or 0.37 to 3.1 in patients who showed renal failure). The same criteria were applied when the IF results were associated with a discordant positive test (vs. the original M-component isotype) during follow-up (oligoclonal bands) [19].

Minimal Residual Disease (MRD) assessment: An NGF-MRD assay was performed on BM aspiration samples (collected in tubes containing EDTA as an anticoagulant) collected from all patients with MM included in the study. For the MRD evaluation, the EuroFlow bulk-lysis and cell surface membrane and cytoplasmic lyse-and-stain standard operating procedures (SOPs) were used, in combination with a two-tube 8-color (10-antibody reagent) EuroFlow NGF-MRD antibody panel (tube 1: CD138 CD27 CD38 CD56 CD45 CD19 CD117 CD81; tube 2: CD138 CD27 CD38 CD56 CD45 CD19 CyIgκ CyIgλ) [20]. For each BM sample, ≥10^7^ stained cells were measured in a FACSCanto II flow cytometer—Becton Dickinson (BD) Biosciences, San Jose, CA—using FACS Diva software (BD). For a data analysis, Infinicyt software (version 2.0, Cytognos SL, Salamanca, Spain) was used. The limit of detection (LOD) of the NGF-MRD method was calculated as 20 cPC/total number of viable cells measured × 100, and the limit of quantification (LOQ) was calculated as 50 cPC/total number of viable cells × 100 [21]. The samples were considered hemodiluted if mast cells were ≤0.002% of the total BM cells, as previously described [20,22].

Statistical analyses: For all statistical analyses, SPSS software (version 21; IBM. Chicago, IL, USA) was used. The nonparametric Mann–Whitney U test was used to establish the statistical significance of the differences observed among groups for unpaired continuous variables. The chi-square test was applied for comparisons between two groups for categorical variables. The Kaplan–Meier method was used to plot survival curves, and the (two-sided) log-rank test was employed to compare PFS and OS curves (both for all patients with MM and for VGPR and CR cases separately). PFS and OS were defined as the time lapse from diagnosis to either disease progression or death by any cause or to the last follow-up visit. For multivariate analyses, the Cox regression model was used to identify variables with an independent prognostic impact on PFS. *p*-values < 0.05 were considered statistically significant.

Ethics: All patients provided written informed consent prior to entering the study, after the study had been approved by the institutional review board.

## 3. Results

Patient characteristics and response to therapy: Overall, 53 patients with MM— with a median age of 58 years (range: 40–70 years; 51% women)—were studied. According to the Durie–Salmon (DS) staging system, most patients (*n* = 36, 68%) were in DS stage III, while their distribution according to the International Score System (ISS) was as follows: stage I, 21 patients (40%); stage II, 17 patients (32%); and stage III, 15 patients (28%). The clinical and demographic features of the patients with MM, stratified according to maintenance therapy, are shown in Table 1, while in Supplementary Table S1, the same features are shown for the whole cohort without stratification. As displayed in Supplementary Table S2, no significant differences were found between the clinical characteristics at diagnosis of the patients treated in the public health system versus those treated in the private health system, except for a greater predominance of more advanced higher ISS stages in the patients from the public health system (*p* = 0.03). At Day + 100 after ASCT, more than half of the patients were in CR (27/53, 51%), of whom a major fraction had also reached sCR (21/53, 40%). In the remaining cases, 21/53 (40%) were in VGPR and 5/53 (9%) in PR. As induction treatment, 27/53 patients (51%) had received CTD, and 26/53 (49%) received VCD, with CR/sCR rates of 48% (13/27) vs. 54% (14/26), respectively (*p* = 0.44). In turn, sCR was achieved in 37% (10/27) of patients treated with CTD vs. 42% (11/26) of those treated with VCD (*p* = 0.44). In addition, PR (7%, 2/27 vs. 12%, 3/26; *p* = 0.66) and VGPR (45%, 12/27 vs. 34%, 9/26; *p* = 0.57) were achieved in similar percentages of cases among patients who had received CTD vs. VCD, respectively.

**Figure 1:**
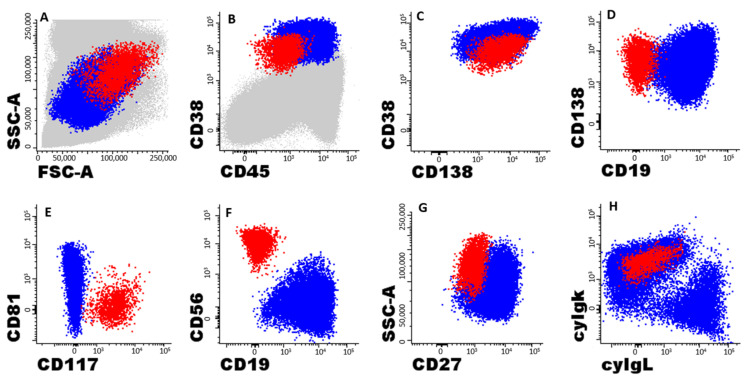
**Illustrative example of the gating strategy used for the identification of residual clonal/aberrant plasma cells by next-generation flow**. Panels show an illustrative example of a patient with multiple myeloma (MM) with minimal residual disease (MRD)-positive bone marrow (BM), in which clonal PCs (cPCs) depicted in red co-exist with a great majority of normal plasma cells (nPCs) depicted as blue dots; PC populations were identified as CD38^hi^ and CD138^+^ cells (panel **C**); other BM cells are shown as gray dots in panels (**A**,**B**). Panel (**A**) shows the light scatter pattern of PCs, in which cPCs show abnormally higher FSC and SSC values than nPCs. As shown in the following panels, cPCs had aberrantly lower expressions of CD38, CD45 and CD27 than nPCs (panels **B**,**G**). In turn, CD19 and CD81 were completely lost in cPCs compared to nPCs (panels **D**–**F**), the former also showing aberrant expressions of CD56 and CD117 (panels **E**,**F**). In addition, cPCs had a restricted expression of intracellular immunoglobulin light chain kappa (CyIgk), while nPCs had a normal CyIgk:CyIgLambda (CyIgL) ratio of 1.5:1 (panel **H**).

Minimal residual disease status at Day + 100 determined by using next-generation flow cytometry: NGF was successfully performed in all 53 patients, and none of the BM samples were inadequate or insufficient for analyses. Flow cytometry studies reached very high sensitivity levels, with a median LOD and LOQ systematically <10^−5^—a median of 0.0002% (range: 0.0001–0.0015%) and of 0.0006% (range: 0.0004–0.0037%), respectively. Out of all 53 BM samples investigated, 32 (60%) were MRD^+^ and 21 (40%) had undetectable MRD. In 10/53 samples (19%), low mast cell counts suggesting BM hemodilution were observed, which included 4/21 (19%) MRD-negative (MRD^−^) samples and 6/32 (19%) MRD^+^ specimens (*p* = 0.62) (Figure 1).

A total of 31/53 (58%) cases showed concordant results between the serologic protein measurement techniques (IF and sFLC) and BM MRD, of which 16/31 (51%) were found to be positive using both methods, and 15/31 (48%) were negative. Among the MRD^−^ cases (6/53, 11.3%), some had a positive IF (4/53, 7.5%) or sFLC (2/53, 3.8%); none of these 6 discrepant cases had IF+ and sFLC+ simultaneously. Conversely, among the patients who were MRD^+^ (16/53, 33%), some had a negative IF (4/53, 7.5%) or sFLC (4/53, 7.5%) or both (8/53, 15.1%), as shown in Supplementary Tables S3 and S4.

**Figure 2:**
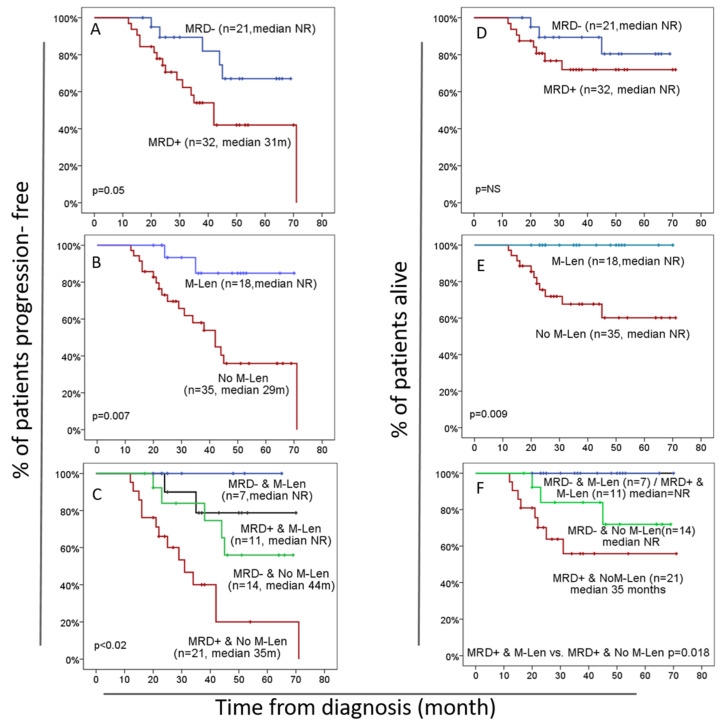
**Kaplan–Meier progression-free survival (PFS—panels A–C) and overall survival (OS—panels D–F) curves of patients with multiple myeloma (MM) submitted to autologous stem cell transplantation (ASCT) and grouped according to lenalidomide maintenance (yes vs. no) and/or bone marrow MRD (MRD^+^ or MRD^−^)**. PFS was significantly lower in patients with MM who had minimal residual disease (MRD)-positive BM (*n* = 32) at Day + 100 after ASCT vs. MRD^−^ cases (*n* = 21), with median progression-free survival of 31 months vs. not reached (panel **A**), respectively; no significant differences in OS were observed between these two patient groups (panel **D**). Patients receiving lenalidomide maintenance (M-Len) after ASCT (*n* = 18) showed significantly better PFS and OS than patients who did not receive maintenance therapy (*n* = 35), with median PFS and OS rates of not reached (NR) vs. 29 months and of NR vs. NR, respectively (panels **B**,**E**). Finally, patients with an MRD^+^ BM who did not receive M-Len (*n* = 21) had significantly shorter median PFS (35 months) and OS (35 months) rates than the other patients (MRD^−^ without M-Len use—*n* = 14; MRD^+^ with M-Len use—*n* = 11; MRD^−^ with M-Len—*n* = 7). In panel (**F**), OS was equal for both groups using M-Len, independently of MRD status; thus, MRD^+^ M-Len and MRD^−^ M-Len curves overlap (panels **C**,**F**).

Impact of the MRD status and lenalidomide maintenance therapy on patient outcome: After a median follow-up of 34 months from diagnosis, disease progression occurred in 21/53 (40%) patients, of whom 5/21 (24%) were MRD^−^, and 16/32 (50%) were MRD^+^ cases (*p* = 0.05), with the median PFS rates post-transplant not reached (NR) vs. 31 months, respectively ([HR 2.62 (95% CI: 0.94–7.29)], *p* = 0.05). Furthermore, 2/5 cases in the MRD^−^ patient group that showed disease progression had an isolated extramedullary relapse, and in 1/5, the BM sample showed signs of being a hemodiluted sample. The median OS was not reached for any of the two MRD^−^ and MRD^+^ patient groups (NR vs. NR; *p* = 0.31) (Figure 2A,D). Similar results were observed when we excluded patients with MM that did not reach VGPR or CR (5/53): disease progression was found in 19/48 (40%) of these latter patients, of whom 5/20 (25%) were MRD^−^ and 14/28 (50%) were MRD^+^, with the median PFS rates not reached (NR) vs. 34 months, respectively (*p* = 0.08). The median OS was not reached for either group (*p* = 0.29).

M-Len therapy after ASCT was used in 18/53 (30%) MM cases, with a median time of therapy of 20.5 months. In this group, only 2/18 patients (11%) experienced disease progression compared to the 19/35 who did not use M-Len (54%), with median PFS rates of NR vs. 29 months, respectively (*p* = 0.007) [HR 5.78 (95% CI: 1.34–24.95)]. Of note, no deaths occurred in the group that received M-Len, while 11/35 (31%) of the patients who did not receive M-Len died, leading to significantly different median OS rates for these two groups (*p* = 0.009) (Figure 2B,E). Among the patients with MM who did not receive M-Len, 15/35 (43%) used thalidomide maintenance, and 20/35 (57%) did not receive maintenance. Notoriously, all patients who were MRD^−^ and showed disease progression did not receive M-Len. PFS and OS analyses showed no significant differences in survival between these two MM patient subgroups (a median PFS of 42 vs. 38 months, *p* = 0.44, respectively, and a median OS of 37 vs. 31 months *p* = 0.11, respectively). More detailed data on the demographics and clinical characteristics of the patients included in the M-Len and no M-Len groups are shown in Table 1.

Of note, the patients who had received M-Len had similar MRD^+^ rates to those who did not receive M-Len: 61% (11/18 patients) vs. 60% (21/35 patients) of MRD^+^ cases (*p* = 0.58). In spite of this, while none of the patients using M-Len who were MRD^−^ had shown disease progression, among the patients who were MRD^−^ who did not receive M-Len, disease progression was found in 43% of cases (*p* = 0.13). Furthermore, among the MRD^+^ cases, significantly different median PFS rates were found depending on whether the patient had used M-Len (NR vs. 35 months, respectively; *p* = 0.011). This also translated into an improved median OS among the patients who underwent M-Len vs. those who did not (NR vs. 35 months, respectively; *p* = 0.018), with no events among the former group of patients (Figure 2C,F).

Univariate and multivariate analyses of prognostic factors for PFS and OS: A univariate analysis of prognostic factors performed based on well-established prognostic factors (age, DS and ISS stages, CR status, the type of induction treatment, MRD status at Day + 100 and the use of M-Len therapy) revealed that only the MRD status at Day + 100 post-ASCT and the use of M-Len therapy had an impact on the PFS of our patients with MM. MRD^−^ vs. MRD^+^ cases showed median PFS rates of NR vs. 31 months [HR 2.62 (95% CI: 0.94–7.29); *p* = 0.049], while patients treated with M-Len vs. those who had no M-Len displayed median PFS rates of NR vs. 29 months [HR 5.78 (95% CI: 1.34–24.95); *p* = 0.003], respectively. A subsequent multivariate analysis showed that both variables (MRD status and M-Len) were independent prognostic factors for PFS in MM, with HRs of 3.37 ((95% CI: 1.19–9.57); *p* = 0.014) and 7.05 ((95% CI: 1.6–30.72); *p* = 0.001) for patients who were MRD^+^ and those who did not receive M-Len, respectively. When we grouped our patients according to both variables, the median PFS rates of NR, NR, 44 months and 35 months were found for MRD^−^/M-Len^+^, MRD^+^/M-Len^+^, MRD^−^/M-Len^−^ and MRD^+^/M-Len^−^ patients, respectively. This was associated with adverse HRs (95% confidence interval) of 2.98 (0.58–15.4) (*p* = 0.19) and 9.22 (2.06–41.2) (*p* = 0.004) for cases that did not receive M-Len and had an MRD^−^ BM and patients who were MRD^+^, respectively (Table 2).

## 4. Discussion

In recent decades, the treatment of MM has dramatically changed due to the introduction of novel agents in combination with new drug combinations and therapeutic schemes [1,23], frequently led by the BM MRD status. This was also associated with the improved monitoring of therapy based on newly developed highly sensitive MRD techniques [3,6]. However, the incorporation of the new drugs/treatment strategies and MRD technologies by low–middle income countries has been challenging and frequently delayed, particularly in public healthcare systems [15]. In addition, most data reported in the literature have been generated in the settings of national protocols or industry-sponsored clinical trials, resulting in limited information about the value of novel therapies and MRD monitoring technologies in real-world patient care, particularly in countries with drug access constraints. Here, we investigated the benefits of new maintenance therapies (M-Len) and highly sensitive MRD measurements in a real-world patient cohort treated in two different healthcare environments in Brazil.

Overall, our findings in a real-world cohort of patients with MM confirm previous results reported in the literature based on clinical trial settings regarding the prognostic benefits on the patient outcome of both the therapy administered (i.e., M-Len) and the BM MRD status achieved with it [6,10,12,24,25]. To the best of our knowledge, this is the first report using NGF for the MRD monitoring of therapy in MM in Latin America and one of the first real-world patient studies using such a treatment monitoring strategy [26]. In 2019, Terpos et al. first reported the monitoring of MRD using NGF as an independent prognostic factor in real-world patients with MM from Greece, outside of clinical trials [26]. Here, we confirm these findings and extend them by also demonstrating a significant benefit in terms of PFS and OS for patients that had access to M-Len therapy compared to those that did not have access to this drug in the ASCT settings, highlighting the need for its fast approval by the public healthcare system. Of note, the few patients treated in the private healthcare system that did not use M-Len due to a lack of approval by the insurance company showed similar results to the patients from the public healthcare system, with a significantly shorter PFS (data not shown).

Even though most patients in our cohort had been diagnosed at (more) advanced stages of the disease compared with other cohorts [27], still, half of them reached CR at Day + 100 following ASCT, in line with previous findings [24]. Despite this, the response did not (significantly) depend on whether they had received VCD or CTD as induction therapy or according to whether they had access to proteasome inhibitors, since only a tendency towards a better outcome among the latter group was observed, in line with other previous reports [28,29]. Interestingly, in our cohort, ISS did not emerge as a relevant prognostic factor for PFS in the univariate analysis, which could be related to the relatively low number of patients in our study; the use of different maintenance regimens in different patients; and the high frequency of stage II/III cases, particularly among patients with MM treated in the public healthcare system. Extending our small cohort with a larger number of patients, preferably in a multicentric setting, would help to confirm the benefit of the inclusion of PI in the regimens used for induction therapy in our real-world settings and to confirm the prognostic impact of ISS.

Regarding the NGF-MRD technique, here, we showed that the implementation of standard EuroFlow procedures and antibody panels in our environment in Brazil provided results highly comparable to those reported by other laboratories [20,30]. This included an easily reachable sensitivity threshold of 2 × 10^−6^ (far beyond the IMWG Flow-MRD threshold criteria of 10^−5^) in virtually every MM case, based on the measurement of very high numbers of cells as recommended by EuroFlow (i.e., ≥10^7^ cells) [20]. From a clinical point of view, MRD undetected by NGF was associated with a significantly better outcome, independently of therapy and other well-established prognostic factors. Furthermore, a similar impact of MRD on PFS and OS was observed when we restricted our analyses to VGPR and CR cases, although the differences did not reach statistical significance, probably due to the small number of patients.

Overall, these results are fully in line with previous findings in the settings of clinical trials, as well as in the limited real-world patient series reported in the literature in which MRD was investigated by using NGF in the BM of treated patients with MM [26]. The increased sensitivity of NGF-MRD compared to that of the consensus 10^−5^ IMWG threshold might be associated with an even higher probability of longer-term disease control, as pointed out by other authors who highlighted the benefit of achieving MRD negativity below the 10^−6^ vs. <10^−5^ thresholds, as reflected by a lower risk of disease progression of patients below vs. above the former threshold [3,31,32]. In turn, this higher sensitivity might contribute to explaining the relatively high rate of discordant results observed in our study with serum protein measurements by, e.g., IF and sFLC, with a greater fraction of NGF-MRD^+^ but IF^−^ and FLC^−^ cases. Despite this, it should be noted that, still, there was a fraction of patients who tested positive using IF or sFLC while NGF-MRD^−^. This might be due to the persistence of the monoclonal protein in serum, despite the clearance of cPCs in BM, as suggested previously [19].

In the few MRD^−^ cases that relapsed, conducting complementary PET-CT imaging to search for extramedullary disease (EMD) (which could not be systematically performed here due to financial constraints) might help to explain our apparently discordant findings, at least in a subset of patients. Such discrepant MRD^−^ results could be explained by a series of factors, such as a lack of M-Len maintenance, extramedullary relapse without BM involvement or sample hemodilution [31,32]. Although we do not have an explanation for two out of five patients who relapsed despite being MRD^−^ at Day + 100, the longer time interval between the MRD assessment and relapse and/or the possibility for a patchy distribution of clonal PCs in the BM at the time of the MRD assessment might also contribute to explaining such apparent discrepancies. In such cases, these false negative MRD results in BM could be mitigated via sequential MRD analyses and/or M-Len therapy.

In addition to the small cohort, our study has two other important limitations: (1) the heterogeneity of the treatment induction regimens administered to the patients, which reflects real life conditions, and (2) the evaluation of MRD at a single time point (Day + 100 after ASCT). In this regard, it has previously been shown that some patients who tested MRD^+^ might convert to MRD^−^ under maintenance therapy with lenalidomide, while others may lose their MRD-negative status, with such kinetics showing (a favorable vs. unfavorable) an impact on patient outcome among those who initially tested as being MRD+ and MRD-, respectively [33,34]. Thus, in future validation MRD studies, a sequential evaluation in larger and more homogeneous patient cohorts is recommended.

Despite all the above limitations of our study, the MRD evaluation carried out using NGF at Day + 100 following ASCT emerged as a powerful prognostic factor, independently of other prognostic factors, including the therapeutic regimen administered. Altogether, these findings support the use of NGF-MRD for the re-assessment of patient risk after therapy (i.e., ASCT) for an improved therapeutic management of MM, as well as in our real-world patient settings.

In addition to MRD, M-Len also emerged as an independent predictor of improved patient outcome. Four randomized studies examined lenalidomide maintenance versus placebo or no maintenance. A meta-analysis conducted on three of these studies [17] and the Myeloma XI trial that was reported separately all provide evidence for a benefit of M-Len [35]. However, in Latin America in general and in Brazil in particular, the incorporation of this drug into the armamentarium of anti-myeloma therapies has been delayed (i.e., Brazil’s recent approval). Because of this, the great majority of patients treated in the public healthcare system environment in Brazil had no access to the drug. Consequently, they did not receive maintenance therapy or just had a short course of thalidomide therapy (based on the gratuity of this latter drug) with some benefit on PFS, but at the expense of treatment discontinuation in cases of neuropathy [15,36]. Here, we report for the first time on the use of M-Len post-ASCT in a cohort of patients treated in the private healthcare insurance system in Brazil. Despite the limited number of patients, our results clearly show a benefit of M-Len in both the PFS and OS of patients with MM who had received ASCT, independently of their MRD status. These results support the well-known immunomodulatory effect of the maintained administration of lenalidomide in sustaining, or even deepening, the response and delaying relapse in MM [17]. Of note, such benefit was independent of the type of induction therapy received by the patients (VCD or CTD), and it was particularly significant among patients who were still MRD^+^ after transplantation. These results are in line with previous findings suggesting that omitting this drug in patients with standard-risk cytogenetics makes them have similar outcomes to patients with high-risk myeloma [35].

To guarantee essential anti-cancer drug access in providing the best standard of care therapy to patients is a well-known universal concern, and it still remains a challenge in practice in the public healthcare systems in Brazil and Latin America. This is mainly due to the higher costs of novel agents often used in combinations and/or administered continuously for long periods of time [27,37]. In this study, we compared for the first time the outcomes of two distinct patient cohorts recruited and treated in parallel with the corresponding standard of care therapies in the public (CTD-ASCT-CTD +/− thalidomide) vs. private insurance (VCD-ASCT-Len) healthcare system environments. Our results show a significant advantage (with regard to both PFS and OS) for patients with supplementary health insurance. In these settings, our data indicate that, in our real-world cohort of patients with MM, the different triplets used as induction therapy prior to ASCT had a relatively limited impact on patient outcome compared to M-Len, with the latter emerging as the strongest independent predictor of patient outcome. Moreover, the combination of M-Len with undetected MRD at Day + 100 following ASCT identified a subset of patients with MM with very good (medium-term) outcomes, particularly when compared to patients who were MRD^+^, did not receive M-Len and had a significantly higher risk of (early) relapse (median PFS of 16 months). Such PFS was less than that described in clinical trials or real-world studies with VCD or lenalidomide, bortezomib and dexamethasone (RVD) as induction therapy (50 to 65 months) [4,5,26,38].

Overall, the relevance of our preliminary data is of utmost importance, since it is estimated that 70% of patients with MM in Brazil to up to 90% in LATAMC are covered by the national public healthcare assistance, pointing out the need for the urgent implementation of policies and measures that will guarantee the human basic principles and rights of equity [15,28]. In this regard, it should be noted that the national drug agencies have already approved the use of both bortezomib and lenalidomide for MM. Therefore, broader access to these (and also other new) drugs requires awareness and active efforts and adoption policies by local public health boards and governmental institutions, in collaboration with national and international medical (i.e., hematology) societies, including guidelines based on the use of drugs included in the WHO list of essential medicines [39]. Thus, negotiation among governments, insurance companies and the pharmaceutical industry, with the possibility for the local production of the drug or biosimilars, is a relevant issue to be urgently addressed for an adequate balance between access to new essential drugs and limited use and, therefore, the benefit of expensive treatments that more fragile economies cannot afford [15,37,40].

## 5. Conclusions

In real-world patients with MM treated in Brazil, the introduction of M-Len post-ASCT is associated with significantly improved survival outcomes, with MRD monitoring via NGF emerging in these settings as a robust and powerful tool to identify subsets of patients with different (higher vs. lower) risks of early relapse and for anticipated treatment decisions. In addition, our data show that the inequity in drug access still remains a hurdle in countries with economic constraints, particularly in the public healthcare system, which has a negative impact on the survival of patients with MM.

## Figures and Tables

**Table 1:** Demographics and baseline clinical and laboratory characteristics of patients with multiple myeloma included in this study grouped according to maintenance therapy (M-Len vs. no M-Len).

Variables Studied at Diagnosis	M-Lenalidomide *n* = 18	No Lenalidomide *n* = 35	*p*-Value
**Age** (years)	57.5 (40–67)	59 (43–70)	0.56
Gender * (% female)	61% (11/18)	45.7% (16/35)	0.22
Subtype of MM *			0.90
**IgG**	67% (12/18)	63% (22/35)	
**IgA**	11% (2/18)	17% (6/35)	
**LC**	17% (3/18)	17% (6/35)	
**NS**	5% (1/18)	3% (1/35)	
**Monoclonal component** (serum)	1.40	2.50	0.77
g/dl	(0–11)	(0–10.1)	
**Monoclonal component** (urine)	0.80	0.85	0.74
g/24 h	(0.37–6)	(0–15.8)	
**Hemoglobin** g/L	115 (69–146)	100 (49–152)	0.18
**Creatinine** mg/dl	0.8 (0.6–5.2)	0.9 (0.5–8.6)	0.16
**Calcium** mg/dl	9.4 (7.7–17)	9.5 (8–14)	0.98
Bone Lesions *	94% (17/18)	91% (32/35)	0.58
DS Stage *			0.14
**II-A and II-B**	44% (8/18)	26% (9/35)	
**III-A and III-B**	56%(10/18)	74% (26/35)	
**ISS Stage**			0.23
**I**	56% (10/18)	31.5% (11/35)	
**II**	22% (4/18)	37% (13/35)	
**III**	22% (4/18)	31.5% (11/35)	
**Albumin** g/dl	3.8 (1.9–6.6)	3.7 (1.4–5.0)	0.16
**Beta2-microglobulin** mg/L	3.1 (1.8–11.3)	3.6 (1.1–33.3)	0.88
Induction treatment *			*0.001*
***CTD***	17% (3/18)	69% (24/35)	
***VCD***	83% (15/18)	31% (11/35)	
Response after ASCT *			0.42
**CR and sCR**	56% (10/18)	49% (17/35)	
**VGPR and PR**	44% (8/18)	51% (18/35)	
**MRD**			0.59
**MRD−**	39% (7/18)	40% (14/35)	
**MRD+**	61% (11/18)	60% (21/35)	

Results expressed as median (range) values or as * number of cases/total cases (percentage). LC, light chain; NS, non-secretory; DS, Durie–Salmon stage; ISS, International Staging System; CTD, cyclophosphamide, thalidomide and dexamethasone; VCD, bortezomib, cyclophosphamide and dexamethasone. The patient group without maintenance with lenalidomide (*n* = 35) included patients who received thalidomide maintenance (*n* = 15) and those who did not receive it (*n* = 20).

**Table 2:** Univariate and multivariate analyses of prognostic factors for progression-free survival (PFS) of patients with multiple myeloma (*n* = 53).

	Univariate Analysis				Multivariate Analysis		
	Median PFS (Months)	HR	95th CI	*p*-Value	HR	95th CI	*p*-Value
**Age at diagnosis**							
<58	37	1					
≥58	28	1.7	(0.69–4.37)	0.23			
**DS**							
II-A	38	1					
II-B	NR	0.34	(0.06–2.06)	0.86			
III-A	27		(0.08–7.88)				
III-B	31		(0.49–5.99)				
**ISS**							
I	36	1					
II	23	0.79	(0.25–2.46)	0.44			
III	35		(0.54–4.51)				
**Induction therapy**							
CTD	34	1					
VCD	35	1.99	(0.79–4.99)	1.13			
**Maintenance therapy**							
No	29	1					
Yes	NR	5.78	(1.34–24.95)	0.003	7.05	(1.6–30.72)	0.001
**Status post ASCT**							
CR	44	1					
Non-CR	30	4.69	(0.18–1.17)	0.10			
**MRD**							
Positive	42	1					
Negative	NR	2.62	(0.94–7.29)	0.049	3.37	(1.19–9.57)	0.014
**MRD and M-Len**							
MRD^−^ or ^+^ and MLen+		1					
MRD^−^ No MLen	44	2.98	(0.58–15.4)	0.19			
MRD^+^ No MLen	35	9.22	(2.06–41.2)	0.004			

CI: confidence interval, HR: hazard ratio, ISS: International Staging System; DS: Durie–Salmon stage, CR: complete response; M-Len: lenalidomide maintenance, No M-Len: no lenalidomide maintenance.

## Data Availability

The data present in this study are available upon request from the corresponding author.

## Supplementary Materials

The following supporting information can be downloaded at https://www.mdpi.com/article/10.3390/cancers15051605/s1, Supplementary Table S1: Demographics and baseline clinical and laboratory characteristics of patients with multiple myeloma included in this study (*n* = 53); Supplementary Table S2: Distribution of clinical features and treatment regimens of patients with multiple myeloma grouped according to the healthcare (public vs. private) environment in which they were treated; Supplementary Table S3: Concordance among MRD status and serologic protein measurements (free light-chain and immunofixation techniques) in patients with multiple myeloma studied at Day + 100 after ASCT (*n* = 53); Supplementary Table S4: Data on the disease status obtained for each individual patient with multiple myeloma included in this study (*n* = 53) by using next-generation flow-MRD, serum-free light-chain and immunofixation techniques.
