# Supplementary material for: Lenalidomide Maintenance and Measurable Residual Disease in a Real-World Multiple Myeloma Transplanted Population Receiving Different Treatment Strategies Guided by Access to Novel Drugs in Brazil
Source: Cancers (Basel). 2023 Mar 4;15(5):1605. doi: 10.3390/cancers15051605 (PMC10000507; doi:10.3390/cancers15051605)
Supplement: Supplementary file 1 [file cancers-15-01605-s001.zip › cancers-2226259-supplementary.pdf]

Supplementary Table S1: Demographics and baseline clinical and laboratory characteristics of patients with multiple myeloma patients included in this study (n = 53)

Patient features	Patient distribution
**Age** (years)	58<br>(40-70)
**Gender*** (% female)	51%<br>(27/53)
**Subtype of MM ***	
**IgG**	64% (34/53)
**IgA**	15% (8/53)
**LC**	17% (9/53)
**NS**	4% (2/53)
**Monoclonal component** (serum)<br>(g/dl)	1.96<br>(0-11)
**Monoclonal component** (urine)<br>(g/24hours)	0.80<br>(0-15.8)
**Hemoglobin**<br>(g/L)	105<br>(49-152)
**Creatinine**<br>(mg/dl)	0.9<br>(0.5-8.6)
**Calcium**<br>(mg/dl)	9.5<br>(7.7-17)
**Bone Lesions***	93% (49/53)
**DS Stage***	
**II-A and II-B**	32% (17/53)
**III-A and III-B**	68% (36/53)
**ISS Stage**	
I	40% (21/53)
II	32% (17/53)
III	28% (15/53)
**Albumin**<br>(g/dl)	3.8<br>(1.4-6.6)
**Beta2-microglobulin**<br>(mg/l)	3.2<br>(1.1-33.3)
**Induction Treatment ***	
CTD	51% (27/53)
VCD	49% (26/53)
**Lenalidomide maintenance***	34% (18/53)

Results expressed as median (range) values or as* percentage (number of cases/total cases) LC: light chain; NS: non-secretory; DS: Durie Salmon stage; ISS: International Staging System; CTD: cyclophosphamide, thalidomide and dexamethasone; VCD: bortezomib, cyclophosphamide and dexamethasone.

Supplementary Table S2: Distribution of clinical features and treatment regimens of patients with multiple myeloma patients grouped according to the healthcare (public vs. private) environment in which they were treated

	**Public Healthcare System** (n=32)	**Private Healthcare System** (n=21)
**Treatment**		
CTD + Thalidomide	24/32 (75%)	0/21 (0%)
VCD + Lenalidomide	0/32 (0%)	15/21 (71%)
VCD	5/32 (16%)	6/21 (29%)
CTD + Lenalidomide[#]	3 /32 (9%)	0/21 (0%)
**Age (Years)**[*]	57 (43-68)	58 40-70
**DS**		
II-A	7/32 (22%)	8/21 (38%)
II-B	1/32 (3%)	1/21 (5%)
III-A	18/32 (56%)	10/21 (48%)
III-B	6/32 (19%)	2/21 (9%)
**ISS**[♥]		
I	8/32 (25%)	13/21 (62%)
II	13/32 (41%)	4/21 (19%)
III	11/32 (34%)	4/21 (19%)

Results expressed as number of cases / all cases in the healthcare group or * as median values (range). ♥*p-value*=0.03. CTD-cyclophosphamide, thalidomide and Dexamethasone; VCD-bortezomib, cyclophosphamide and dexamethasone; DS-Durie Salmon stage; ISS- International Staging System.

Supplementary Table S3: Concordance among MRD status and serologic protein measurements (free -light- chain and immunofixation techniques) in patients with multiple myeloma patients studied at Day + 100 after ASCT (n = 53)

Accordance	MRD *status*	FLC	IF	N. of Cases	% of Cases
**Concordant cases**	+	+	+	16	30.2%
	-	-	-	15	28.3%
**(subtotal)**				**31**	**59%**
**Discordant cases**	+	-	+	4	7.5%
		+	-	4	7.5%
		-	-	8	15.2
	-	-	+	4	7.5%
		+	-	2	3.8%
**(subtotal)**				**22**	**41%**

+: Positive, -: Negative, MRD: measurable residual disease, FLC: free-light chain, IF: immunofixation, Concordant cases: concordant results between MRD, FLC and IF, Discordant cases: considering the MRD status as the golden-standard criteria to define MM treatment response, thus negative results of FLC and/or IF compared to an MRD+ status were considered as "False Negative". Conversely, positive results of FLC and/or IF compared to MRD- status, were considered as "False Positive".

Supplementary Table S4: Data on the disease status by next generation flow-MRD, serum free light chain and immunofixation techniques obtained for each individual patient with multiple myeloma patient included in this study (n = 53) by using next-generation flow-MRD, serum-free light-chain and immunofixation techniques.

Case	Induction Therapy	M-component subtype	NGF	Hemodilution	FLC ratio	IF	Oligoclonal bands
			**Discordant cases**				
1	VCD	IgG-L	Negative	yes	1,01	Positive	No
2	VCD	IgA-K	Negative	No	1,26	Positive	No
3	CTD	IgG-K	Negative	No	2,50	Negative	Yes
4	VCD	Kappa	Negative	No	0,83	Positive	No
5	CTD	IgG-L	Negative	No	1,89	Negative	No
6	CTD	IgG-L	Negative	No	0,46	Positive	No
7	CTD	IgG-K	Positive	No	2,62	Negative	yes
8	VCD	NS	Positive	No	1,25	Negative	No
9	VCD	IgG-L	Positive	No	0,74	Negative	No
10	VCD	Kappa	Positive	No	1,35	Negative	No
11	CTD	IgA-K	Positive	No	0,67	Negative	No
12	VCD	Kappa	Positive	No	4,94	Negative	No
13	VCD	IgG-K	Positive	No	0,93	Negative	No
14	CTD	IgG-K	Positive	No	1,56	Positive	No
15	CTD	IgG-K	Positive	No	2,93	Negative	No
16	VCD	IgG-K	Positive	No	1,95	Negative	No
17	CTD	IgG-K	Positive	No	1,15	Negative	Yes
18	VCD	IgA	Positive	No	1,56	Negative	Yes
19	CTD	IgG-L	Positive	No	0,67	Positive	No
20	CTD	IgG-K	Positive	No	1,09	Positive	No
21	VCD	IgG-K	Positive	No	1,61	Negative	No
22	CTD	IgG-K	Positive	Yes	1,21	Positive	No
			**Concordant Cases**				
24	VCD	IgG-K	Negative	No	0,88	Negative	No
25	VCD	IgA-K	Negative	No	1.1	Negative	yes
26	CTD	IgA	Negative	No	1,28	Negative	No
27	VCD	NS	Negative	No	NM	Negative	No
28	CTD	Lambda	Negative	No	1,89	Negative	Yes
29	VCD	IgG-K	Negative	No	1,47	Negative	Yes
30	CTD	IgA-K	Negative	Yes	1,05	Negative	No
31	CTD	IgG-L	Negative	No	1,33	Negative	Yes
32	VCD	IgG-K	Negative	No	1,13	Negative	No
33	CTD	IgG-L	Negative	No	1,05	Negative	No
34	CTD	IgG	Negative	No	0,98	Negative	No
35	VCD	Kappa	Negative	Yes	1,08	Negative	No
36	VCD	IgG-K	Negative	No	1,40	Negative	No
37	CTD	IgG-L	Negative	No	1,26	Negative	No
38	CTD	Lambda	Negative	Yes	0,54	Negative	No
39	VCD	IgG-K	positive	No	2,15	positive	No
40	CTD	IgG-L	positive	Yes	0,14	positive	No
41	VCD	IgG-K	positive	No	2,83	positive	No
42	VCD	IgG-K	positive	No	1,91	positive	No
43	VCD	IgA-K	positive	No	4,20	positive	No
44	CTD	Kappa	positive	No	7,80	positive	Yes
45	CTD	Kappa	positive	No	26,97	positive	No
46	CTD	IgA-K	positive	No	4,30	positive	No
47	CTD	Lambda	positive	No	0,09	positive	No
48	CTD	IgG-L	positive	Yes	0,01	positive	No
49	VCD	IgG-K	positive	Yes	11,34	positive	No
50	CTD	IgG-K	positive	Yes	29,79	positive	Yes
51	CTD	IgG-L	positive	No	2,02	positive	Yes
23	VCD	IgG-L	Positive	Yes	0,25	Positive	No
52	VCD	IgG-K/L	positive	No	0,23	positive	No
53	VCD	IgG-K	positive	No	5,49	positive	No

CTD: cyclophosphamide, thalidomide, dexamethasone; VCD: bortezomib, cyclophosphamide, dexamethasone, NGF: next generation flow; sFLC ratio: serum free-light chain ratio, IF: immunofixation; NM: not measurable; *Patient with renal insufficiency at diagnosis.
